# Supplementary material for: Antibiotic use in infants within the first year of life is associated with the appearance of antibiotic-resistant genes in their feces
Source: PeerJ. 2023 May 15;11:e15015. doi: 10.7717/peerj.15015 (PMC10194068; doi:10.7717/peerj.15015)
Supplement: Supplemental Information 1 — + antibiotic use 0-12 age in months; - no antibiotic use; -+ no antibiotic use but there is herbal/natural products use; ++ antibiotic use and also herbal/natural product use; * no data collection; VD, vaginal delivery; CS, cesarean section; -+*, ++*, +*, -* sample was not collected. Still, there was no use of antibiotics and natural products ● ---- dfr ● ---- TEM β ----- Erm B Ω ----Mef ● -----SHV ∞ ----Tet A γ----- Aac ● -----CTX-M α----- Erm A µ------ BlaZ [file peerj-11-15015-s001.docx]

**S1 Table:** Occurrence of the resistant genes in relation to antibiotic use

| Babies | 0 | 1 | 2 | 3 | 4 | 5 | 6 | 7 | 8 | 9 | 10 | 11 | 12 |
| --- | --- | --- | --- | --- | --- | --- | --- | --- | --- | --- | --- | --- | --- |
| 1 (CS) | - | - ∞γµΩ | - ∞ γµ | - ∞µ | - ∞βµΩ | - ∞Ω | - ∞ β γµΩ | + ∞ β γΩ | - ∞Ω | - ∞ β γΩ | + ∞ β γΩ | - ∞ β γΩ | - ∞ βΩ |
| 2 (CS) | -Ω | + ∞Ω | -* | * | - | * | * | * | * | * | * | * | * |
| 3(VD) | -● β γµΩ | - ∞ α γµΩ | -● α β γµΩ | -●∞ α β γµΩ | -* | - ∞ βΩ | -* | - ∞Ω | - ∞Ω | - ∞Ω | - ∞Ω | * | * |
| 4(VD) | -● Ω | +* | - ∞β γµ Ω | - ∞ βµ Ω | -* | * | -* | - ∞ β Ω | - ∞ Ω | - ∞ γµ Ω | - ∞ β Ω | * | * |
| 5(VD) | -● β γµ | + | - | -* | -* | - | - | - | - | - | - | * | * |
| 6(CS) | -* | -●● | -* | -* | -* | -* | -* | - | +● | - | * | * | * |
| 7(CS) | +* | - | - | -+ | -+* | - | -* | -+● | -+ | -+ | * | * | * |
| 8(VD) | +● | -● | - | -+● | -+ | -● | -● | - | - | - | - | * | * |
| 9(VD) | - | - | - | - | * | - | * | * | - | - | * | * | * |
| 10(CS) | -● β Ω | -● | - Ω | +● β Ω | -* | - β | - ∞ Ω | - ∞ βγ Ω | + β | +● β | - | * | * |
| 11(CS) | -∞ | -●● γ | -●● β γ | + | -* | - β | - β | - ∞ β | -● ∞ β | + ∞ β | - ∞ β | * | * |
| 12(CS) | + ∞ β Ω | -* | -* | -+* | -+* | * | * | * | * | * | * | * | * |
| 13(CS) | + β γ | -* | -* | * | * | * | * | * | * | * | * | * | * |
| 14(VD) | - β | -* | -* | -* | -* | - ∞ | -* | + α β Ω | - β Ω | + γ Ω | * | * | * |
| 15(CS) | +● β | + β | - β | - | -* | -* | - α | -* | + | - | * | * | * |
| 16(CS) | + Ω | +● γ Ω | -●∞ Ω | +* | -* | -+* | ++* | - ∞ β Ω | - | - βµ Ω | * | * | * |
| 17(CS) | -●∞ γµ Ω | - ∞ β γ | -* | - ∞ β γ | -* | + β γµ | - ∞ β γ | - | - ∞ γµ Ω | * | * | * | * |
| 18(VD) | - γµ | +* | * | * | * | * | * | * | * | * | * | * | * |
| 19(VD) | -* | * | * | * | * | * | * | * | * | * | * | * | * |
| 20(VD) | -●∞ γµ | -●∞ Ω | -* | + β | -* | + ∞ β Ω | + β γµ | -* | -●∞ β γ Ω | * | * | * | * |
| 21(VD) | - | -● | -+● | -+ | -+ β | -+µ Ω | -+ γµ Ω | - γµ Ω | * | * | * | * | * |
| 22(CS) | + β Ω | * | * | * | * | * | * | * | * | * | * | * | * |
| 23(VD) | -µ | -* | -* | -* | -* | -●∞ α β Ω | - ∞ α Ω | - ∞ α Ω | - ∞ α γµ Ω | * | * | * | * |
| 24(VD) | +● γµ | -+ β | - ∞ Ω | -* | - ∞ | - Ω | +●∞ β Ω | - β γµ | * | * | * | * | * |
| 25(VD) | - ∞ Ω | * | * | * | * | - ∞β γ Ω | * | + β γ | -* β γ | - | * | * | * |
| 26(VD) | - β∞ γ Ω | - β∞ γ Ω | -* | -* | - ∞ β Ω | +●∞ β | - ∞ γ | - ∞ β γ | * | * | * | * | * |
| 27(CS) | - ∞ β γ | - ∞ Ω | -● ∞ | -* | - ∞ β Ω | + ∞ β Ω | - β | - | -* | * | * | * | * |
| 28(CS) | - | - | - β | -* | - | + | - | - | + | * | * | * | * |
| 29(VD) | - | * | * | * | * | * | * | * | * | * | * | * | * |
| 30(VD) | - | * | * | * | * | * | * | * | * | * | * | * | * |

+ Antibiotic use 0-12 age in months

- No antibiotic use -+ No antibiotic use but there is herbal/natural products use

++ Antibiotic use and also herbal/natural product use * No data collection

VD vaginal delivery CS caesarean section

● ---- dfr ● ---- TEM β ----- Erm B Ω ----Mef

● -----SHV ∞ ----Tet A γ----- Aac

● -----CTX-M α----- Erm A µ------ BlaZ
